# Multi-trait spectral modeling for estimating grapevine leaf traits and nutrients

**DOI:** 10.1016/j.plaphe.2025.100142

**Published:** 2025-11-17

**Authors:** Parastoo Farajpoor, Alireza Pourreza, Mohammadreza Narimani, Ashraf El-kereamy, Matthew W. Fidelibus

**Affiliations:** a Digital Agriculture Laboratory, Department of Biological and Agricultural Engineering, University of California, Davis, CA, USA; b Department of Botany and Plant Sciences, University of California, Riverside, CA, USA; c Department of Viticulture and Enology, University of California, Davis, CA, USA

**Keywords:** PROSPECT-PRO, Multi-trait modeling, Spectral modeling, Leaf nutrients, Digital viticulture, Grape, Long short-term memory networks, Remote sensing

## Abstract

Analysis of leaf hemispherical radiative properties for retrieval of its biochemical and mineral nutrients could lead to a powerful monitoring approach for precise farm management. This study explores the potential of leaf spectral modeling techniques for estimation of key biochemical and nutritional traits in grapevine leaves. Hyperspectral data spanning the 400–2500 ​nm range were collected from around 1000 leaf grapevine leaf samples across three growing seasons. Certain traits, including leaf structural parameter (Nstruct), anthocyanins, carotenoids, and chlorophyll, were imputed using the PROSPECT-PRO radiative transfer model in the inverse mode to enrich the dataset. An imputation model was developed to address missing labels for part of the dataset, employing a Convolutional Neural Network (CNN) with 23 principal components derived from the spectral data as inputs. This model enabled the completion of the dataset by predicting missing trait values, providing a comprehensive foundation for subsequent modeling efforts. For the primary trait prediction models, the spectral data were then reduced from 2101 bands to 204 bands through band merging based on pairwise correlations. Two predictive modeling approaches were evaluated: a single-trait model, where each trait is predicted independently, and a multi-trait model, where all traits are predicted simultaneously. Both models employed a hybrid of CNN and Long Short-Term Memory (LSTM) networks designed to capture spatial and sequential patterns in spectral data. The single-trait model utilized CNN-LSTM architecture with a single output node, requiring independent training for each trait. In contrast, the multi-trait model employed the same architecture but featured 16 output nodes, enabling the simultaneous prediction of all traits. A weighting strategy was implemented to balance the influence of fully measured and imputed samples during training, ensuring reliable predictions. The multi-trait model demonstrated superior predictive performance across most traits, achieving a higher coefficient of determination (R^2^) and RPD (Residual Predictive Deviation), and lower normalized root mean squared error (NRMSE) values than the single-trait models. Some traits, such as nitrogen, phosphorus, Nstruct, and manganese benefited significantly in the multi-trait model with R^2^ values of 0.42, 0.81, 0.90, and 0.62, respectively, compared to 0.26, 0.64, 0.25, and 0.30 in single-trait models. The results highlight the advantages of multi-trait modeling in leveraging shared spectral information and inter-trait dependencies, offering an efficient and accurate approach to predicting grapevine traits.

## Graphical abstract

**Figure:**
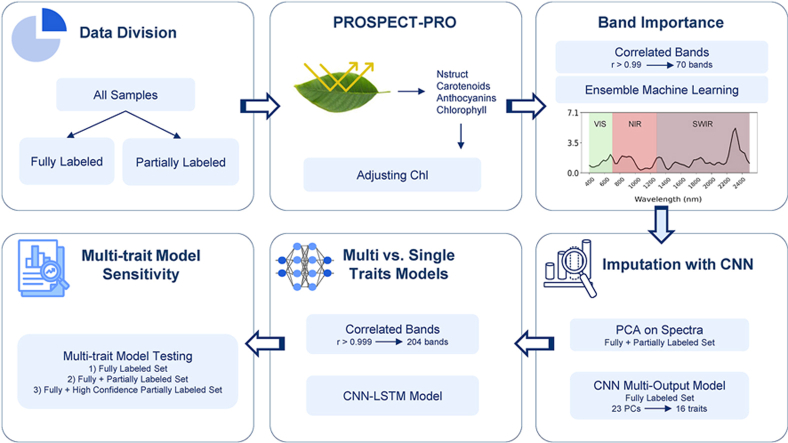

**Table:** Table of abbreviations

Abbreviation	Name
N	Total Nitrogen
P	Phosphorus
K	Potassium
Ca	Calcium
Mg	Magnesium
Zn	Zinc
Mn	Manganese
Fe	Iron
Cu	Copper
B	Boron
Chl	Chlorophyll
Car	Carotenoids
Ant	Anthocyanins
EWT	Equivalent Water Thickness
LMA	Leaf Mass per Area
Nstruct	Leaf Structural Parameter
PCA	Principal Component Analysis
PC	Principal Component
R^2^	Coefficient of Determination
NRMSE	Normalized Root Mean Squared Error
RMSE	Root Mean Squared Error
MSE	Mean Squared Error
RPD	Residual Predictive Deviation
PLSR	Partial Least Squares Regression
RF	Random Forest
XGB	Extreme Gradient Boosting
SVR	Support Vector Regression
CNN	Convolutional Neural Networks
LSTM	Long-Short Term Memory
CV	Cross Validation
RTM	Radiative Transfer Model

## 1 Introduction

Grapes, including raisin, table, and wine varieties, are the most important fruit crops in California, generating $5.5 billion in cash receipts in 2022 [1]. The San Joaquin Valley (SJV) of California is an especially important grape-growing region, producing globally significant crops of table, raisin, and wine grapes. The deep soils of the SJV can provide many of the mineral nutrients required by grapes, but supplemental nitrogen (N), potassium (K), magnesium (Mg), boron (B), and zinc (Zn) are commonly required [2]. A severe deficiency of these nutrients results in distinctive foliar and cluster symptoms [3]. However, by the time clear-cut visual symptoms are noticed, effects on yield and quality may have already been established. Therefore, grape growers generally collect plant tissue samples, petioles, and/or leaf blades at standard phenological stages (bloom and veraison) to monitor vine nutrition. Tissue tests can identify mineral nutrition issues before visual symptoms are observed and thereby help prevent yield or quality loss. They also help prevent unnecessary fertilizer applications, which needlessly increase production costs and could cause unintended nutrient imbalances or environmental contamination. However, collecting tissue samples is costly, labor-intensive, and time-consuming, and the results lack spatial resolution [4]. Technological advancements may enable remote sensing systems to help address many of the challenges associated with field sampling [[5], [6], [7], [8]]. For example, remote sensing has shown the capability to reduce the need for extensive sample collection and minimize the variability introduced by different sampling techniques [9]. Early approaches primarily used spectral data to calculate vegetation indices, such as the normalized difference vegetation index (NDVI), to serve as proxies for plant traits, including nutrient content [10]. However, vegetation indices rely on simple spectral combinations and assume direct correlations with plant traits, whereas complex plant-environment interactions may influence these relationships [[11], [12], [13]].

Consequently, linear approaches such as Partial Least Squares Regression (PLSR) were used to capture spectral-trait relationships. For instance, Helsen et al. [14] employed PLSR to predict leaf mass per area (LMA), leaf dry matter content (LDMC), and leaf water content (EWT) from spectral data, achieving high accuracy (R^2^ ​> ​70 ​%, NRMSE <10 ​%) using species-specific models. Cuq et al. [15] applied PLSR to near-infrared spectral data to predict nutrient concentrations in vine organs, achieving reliable predictions for elements such as calcium (Ca), Mg, K, and Zn with R^2^ values up to 0.88. PLSR provides interpretable predictions due to its linear structure, making it straightforward to understand trait relationships, but it may fail to capture complex, nonlinear interactions that machine learning methods can address more effectively [16,17]. Several studies have used spectral data to either compare PLSR with machine learning techniques or combine them to enhance the accuracy for plant trait estimation [18,19]. For example, Chancia et al. [20] used a machine learning ensemble feature selection approach to identify optimal spectral bands for grapevine nutrient prediction from UAS-based hyperspectral imagery. Their method produced comparable regression accuracy to PLSR with fewer wavelengths. In another study, Peng et al. [21] used PLSR along with machine learning models such as Random Forest (RF), Support Vector Machine (SVM), and Extreme Learning Machine (ELM) to predict N, K, and phosphorus (P) content in grape leaves using UAV-based multispectral imagery. The study found that RF, SVM, and ELM performed better, with R^2^ values above 0.65 during early growth stages and exceeding 0.75 in later stages, indicating that advanced machine learning models provided superior predictive accuracy. Moghimi et al. [22] evaluated various machine learning models for N estimation in grapevines using high-resolution multispectral imagery at the canopy level, achieving promising accuracy of R^2^ ​= ​0.56 and RMSE ​= ​0.23.

Advancements in machine learning and deep learning models have expanded their use in agricultural remote sensing, enhancing their capability to analyze complex data and improve decision-making [[23], [24], [25], [26], [27], [28], [29]]. Among complex machine learning and deep learning models, CNNs stand out in remote sensing for their ability to automatically learn and extract spatial patterns from spectral data [30]. For example, Xi et al. [31] developed a one-dimensional CNN (Conv1D) to classify tree species using OHS-1 hyperspectral imagery, demonstrating higher accuracy (85.04 ​%) compared to the RF model (80.61 ​%), particularly for broadleaf species with similar spectral characteristics. However, while CNNs excel at capturing spatial features in spectral data, they may not fully exploit the sequential dependencies between adjacent wavelengths. Integrating Long Short-Term Memory (LSTM) networks helps address this by modeling spectral continuity and leveraging wavelength correlations, enhancing the predictive power of deep learning models for hyperspectral data [32,33]. For example, Liu et al. [34] developed an LSTM-CNN-Attention model to predict soil properties using spectral and achieved higher accuracy (R^2^ above 0.92) compared to traditional machine learning and deep learning models such as PLSR, Support Vector Regression (SVR), RF, LSTM, and CNN.

Spectral data can be used in computational models to predict plant traits at both the leaf and canopy levels. Canopy-level data, while applicable for large-scale monitoring, can be influenced by factors like environmental conditions and sun angle, requiring careful calibration for accurate predictions [35]. At the leaf level, proximal spectral sensing directly measures the spectral reflectance of individual leaves [36]. This approach captures detailed biochemical and physiological information, including pigments and essential nutrients [37]. However, trait prediction models rely on comprehensive datasets that integrate spectral and plant trait information, which can be inconsistent due to variations in data collection across years, locations, and experimental conditions. For example, certain traits might be measured in one dataset but not in another, while others are consistently recorded. In these scenarios, various imputation methods address inconsistencies in trait availability. These methods range from basic statistical interpolation to advanced machine learning algorithms, ensuring data completeness and enhancing model training for improved generalization across diverse agricultural conditions [38,39].

Furthermore, data-driven models employing spectral data for plant trait prediction often treat traits individually, overlooking their inherent interdependencies. In contrast, multi-trait models leverage these interdependencies to predict multiple traits simultaneously, thereby providing a more holistic understanding of plant biochemistry [[40], [41], [42]]. Such approaches might be particularly beneficial in scenarios where multiple traits simultaneously affect the same spectral region, enabling a more precise separation of the individual impacts of traits on leaf hemispherical radiative properties [43,44].

Aside from data-driven models, Radiative Transfer Models (RTM) offer an alternative approach to trait estimation. Among these, the PROSPECT-PRO model has gained prominence for retrieving leaf biochemical traits from leaf hyperspectral data. Operating in its inverse mode, PROSPECT-PRO provides robust estimates for parameters such as chlorophyll (Chl), anthocyanins (Ant), and carotenoids (Car), leveraging the physical principles of light interaction with leaf structures and pigments [45]. These models are particularly valuable for their mechanistic basis, offering interpretable relationships between spectral data and leaf biochemistry. However, the PROSPECT-PRO model is calibrated using subsets of the LOPEX dataset, which may introduce uncertainty or bias when applied to unseen datasets for specific crops.

The primary goal of this research is to explore advanced modeling techniques and hyperspectral data for estimating grapevine leaf biochemical and nutritional traits to inform vineyard management practices. This study proposes a hybrid LSTM-CNN multi-trait modeling approach to predict 16 leaf traits simultaneously by capturing complex interdependencies within traits. Specifically, the objectives are to 1) implement and evaluate an imputation model to enhance dataset completeness and 2) compare the performance of single-trait and multi-trait predictive models in accuracy and efficiency. This study will evaluate the efficacy of a multi-trait approach as a comprehensive, efficient, and scalable solution for nutrient monitoring in vineyards.

## 2 Materials and methods

### 2.1 Study area and data collection

This study was conducted at the University of California's Kearney Agricultural Research and Extension Center (KREC) in Parlier, California. Data was collected from 2021 through 2023 from three different grapevine varieties: Sunpreme, a white-fruited raisin variety; Flame Seedless, a red-fruited table grape variety; and Solbrio, a black-fruited table grape variety. Flame Seedless was grown in pots, whereas Sunpreme and Solbrio were grown in vineyards. Growing practices for Flame Seedless and Solbrio were described previously [4].

A total of 2305 leaves, representing 992 unique samples, were collected from different vine heights to capture a broad range of leaf biochemical and nutritional properties. To further enhance data diversity, the samples comprised leaves from prebloom (40 samples), bloom (72 samples), berry set (72 samples), veraison (450 samples), and harvest (71 samples), with 287 samples lacking specified stage information. The spectral reflectance of each leaf was measured using the SVC HR-1024i high-resolution field spectrometer (Spectra Vista Corp, NY, USA) in combination with the Leaf Clip and Reflectance Probe. This device integrates a contact probe with an internal tungsten halogen light source, ensuring consistent illumination for accurate reflectance measurements over a spectral range of 350–2500 ​nm. A white Spectralon disk was used for calibration, with reference measurements taken after every five leaf samples to maintain standardized reflectance readings. The spectrometer also included an automated dark current correction to further refine measurement reliability. Previous multi-point measurements across leaf blades showed consistent reflectance, justifying single-point spectral readings on either side of the apical node of each leaf [4].

Following spectral measurements, the fresh weight of each leaf was recorded. Leaf area was determined using a LI-COR leaf area meter, after which leaves were rinsed with deionized water and blotted dry. Approximately 0.1 ​g (fresh weight) of leaf tissue was collected from all the leaves in a sample by punching 10 to 13 discs with a 9 ​mm diameter cork borer to assess Chl content. The discs were stored in microcentrifuge tubes at −20 ​°C until they could be extracted with methanol, following the method of Warren [46]. The remaining tissue was dried in a forced-air oven at 60 ​°C until no further weight loss was observed, then reweighed to determine dry weight. LMA and EWT were subsequently calculated using the following formulas:

(1) $$LMA=\frac{Dry\;weight}{Leaf\;area}$$

(2) $$EWT=\frac{Fresh\;weight-Dry\;weight}{Leaf\;area}$$

The tissue was then finely ground into powder and sent to a commercial laboratory to analyze mineral nutrient content. To standardize the data, all traits were normalized to leaf area, ensuring they were expressed on an area basis. This approach allows for easier scaling of measurements to canopy levels using the leaf area index [47,48].

After completing this process, a comprehensive set of ground truth leaf traits was obtained for each sample, as detailed in Table 1. These traits include N, P, K, Ca, Mg, Zn, manganese (Mn), iron (Fe), copper (Cu), B, Chl, EWT, and LMA.

**Table 1:** Summary statistics of 13 measured grapevine leaf traits in area and mass-based across 12 datasets. #n = Number of samples, Min = Minimum, Max = Maximum, SD = Standard Deviation.

Date	Variety	Stage	#n	Traits												
				N	P	K	Ca	Mg	Zn	Mn	Fe	Cu	B	Chl	EWT	LMA
2021-06-09	Flame Seedless	Veraison	183	**✓**											**✓**	**✓**
2021-07-27	Flame Seedless	NA	287	**✓**											**✓**	**✓**
2022-06-16	Solbrio	Veraison	195	**✓**											**✓**	**✓**
2023-04-27	Solbrio	Pre-bloom	40	**✓**	**✓**	**✓**	**✓**	**✓**	**✓**	**✓**	**✓**	**✓**	**✓**	**✓**	**✓**	**✓**
2023-05-12	Solbrio	Bloom	40	**✓**	**✓**	**✓**	**✓**	**✓**	**✓**	**✓**	**✓**	**✓**	**✓**	**✓**	**✓**	**✓**
2023-05-12	Sunpreme	Bloom	32	**✓**	**✓**	**✓**	**✓**	**✓**	**✓**	**✓**	**✓**	**✓**	**✓**	**✓**	**✓**	**✓**
2023-05-26	Solbrio	Berry set	40	**✓**	**✓**	**✓**	**✓**	**✓**	**✓**	**✓**	**✓**	**✓**	**✓**	**✓**	**✓**	**✓**
2023-05-26	Sunpreme	Berry set	32	**✓**	**✓**	**✓**	**✓**	**✓**	**✓**	**✓**	**✓**	**✓**	**✓**	**✓**	**✓**	**✓**
2023-06-22	Solbrio	Veraison	40	**✓**	**✓**	**✓**	**✓**	**✓**	**✓**	**✓**	**✓**	**✓**	**✓**	**✓**	**✓**	**✓**
2023-06-22	Sunpreme	Veraison	32	**✓**	**✓**	**✓**	**✓**	**✓**	**✓**	**✓**	**✓**	**✓**	**✓**	**✓**	**✓**	**✓**
2023-07-27	Solbrio	Harvest	39	**✓**	**✓**	**✓**	**✓**	**✓**	**✓**	**✓**	**✓**	**✓**	**✓**	**✓**	**✓**	**✓**
2023-07-27	Sunpreme	Harvest	32	**✓**	**✓**	**✓**	**✓**	**✓**	**✓**	**✓**	**✓**	**✓**	**✓**	**✓**	**✓**	**✓**
Total number of samples			992	12	9	9	9	9	9	9	9	9	9	9	12	12

Unit				mg/cm^2^	μg/cm^2^	μg/cm^2^	μg/cm^2^	μg/cm^2^	μg/cm^2^	μg/cm^2^	μg/cm^2^	μg/cm^2^	μg/cm^2^	μg/cm^2^	mg/cm^2^	g/m^2^

Min				0.08	2.35	15.54	21.46	5.88	0.02	0.16	0.13	0.02	0.07	22.07	8.86	35.09
Max				0.32	35.37	92	285.67	47.3	0.54	1.46	9.62	1.96	0.52	73.23	27.74	118.3
Average				0.15	13.5	48.64	115.12	24.38	0.11	0.58	0.62	0.38	0.24	35.88	16.05	61.47
SD				0.03	4.34	12.76	52.15	9.02	0.04	0.28	0.72	0.44	0.06	8.59	1.58	12.77

Unit				%	%	%	%	%	mg/kg	mg/kg	mg/kg	mg/kg	mg/kg	mg/g FW	g	

Min				1.25	0.03	0.15	0.31	0.09	3.51	22.6	19.5	2.35	10.2	1.17	0.27	
Max				4.83	0.71	1.43	3.98	0.71	92	248	1650	396	101	3.71	5.55	
Average				2.56	0.23	0.80	2.15	0.41	19.7	91.8	109.5	66.2	43.5	1.84	2.00	
SD				0.61	0.11	0.28	0.75	0.10	8.59	48.8	120.8	86.5	11.8	0.43	0.88	

### 2.2 Data curing and preprocessing

Linear interpolation was applied to standardize the spectral reflectance values at a 1 ​nm interval across the 400–2500 ​nm range to ensure uniformity in spectral data. This interpolation allowed for consistent comparisons and analyses across all samples, as the original spectral data varied in measurement intervals.

**Fig. 1:**
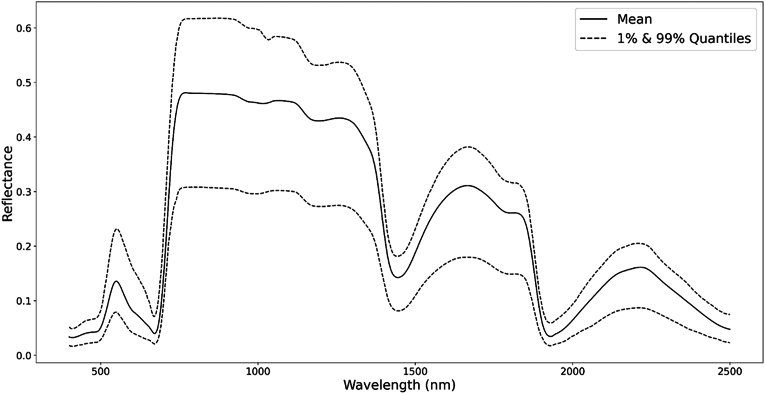
Mean and 1 ​%–99 ​% quantiles of spectral reflectance across all datasets.

Following interpolation, a Savitzky-Golay filter was used to smooth the spectral data and reduce noise. This filter was applied with a window size of 75 ​nm and a polynomial order of 3. The chosen window size and polynomial order allowed for the preservation of spectral features while minimizing data irregularities, thus enhancing the quality and interpretability of the reflectance curves (Fig. 1). This preprocessing ensured the spectral data were optimally prepared for subsequent model development and trait analysis.

### 2.3 Model development

The dataset was divided into two groups to facilitate model training and imputation tasks. The first group, referred to as the “Fully Labeled Set,” contained 327 samples with measurements available for all traits. The second group, described as the “Partially Labeled Set,” included 665 samples with some traits missing, which would later require imputation.

#### 2.3.1 PROSPECT-PRO estimated traits

The ground truth set lacked essential labels, with Nstruct, Ant, and Car missing from all samples, and Chl unavailable for 665 samples in the Partially Labeled Set. To enhance the dataset with these critical leaf traits, which influence the hemispherical radiative properties of leaves, the PROSPECT-PRO model was employed in the inverse mode using optimal spectral bands to estimate their values.

Given that the Chl data comprised both direct measurements and estimates derived from PROSPECT-PRO, it was essential to evaluate the accuracy of the PROSPECT-PRO predictions to ensure data consistency and reliability. A comparison of actual Chl values with PROSPECT-PRO estimated values was conducted on 327 samples of the Fully Labeled Set.

**Fig. 2:**
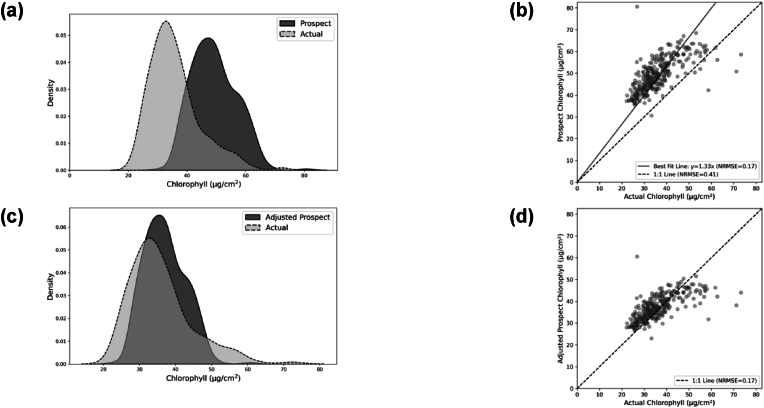
Comparison of PROSPECT-PRO Chl estimates with actual values: (a) and (b) Before adjustment; (c) and (d) After adjustment.

A scaling factor was applied to align the predictions more closely with the actual measurements to correct for potential bias in the PROSPECT-PRO estimates. The scaling factor, calculated as the reciprocal of the slope of the best-fit line in Fig. 2-b, was determined to be 0.75. Subsequently, this scaling factor was applied to the PROSPECT-PRO estimated Chl values of all the samples in the Partially Labeled Set that lacked direct Chl measurements.

For N, LMA, and EWT, PROSPECT-PRO estimated values were also obtained to facilitate a later comparison with the performance of the multi-trait model. Using the protein-to-nitrogen conversion factor (k_p_) of 4.43 [49] N was calculated from the protein predicted by PROSPECT-PRO, while LMA was derived from the sum of predicted protein and CBC (Carbon-Based Content) values.

#### 2.3.2 Inter-trait relationships and significance of wavelengths

During data inspection, it was noted that four samples within the Fully Labeled Set had Fe and Zn concentrations approximately ten times higher than the typical values. This anomaly was likely due to contamination. To maintain data integrity, the Fe and Zn values for these four samples were removed, and the samples were subsequently transferred to the Partially Labeled Set. This transfer allowed for the imputation of the missing Fe and Zn values for these samples in subsequent modeling steps.

To develop a model for imputing leaf traits using spectral data, considering trait relationships was essential for improving generalization and robustness. Using the Fully Labeled Set, we first examined the relationship between pairs of traits using the Spearman correlation as defined in Equation (3) for its ability to measure non-linear associations [50].

(3) $$\rho =1-\frac{6\sum d_{j}^{2}}{n\;\left(n^{2}-1\right)}$$

Where:

• ρ ​= ​Spearman's rank correlation coefficient
• $d_{i}$ ​= ​difference between the two ranks of each observation
• n ​= ​number of observations

Due to differences in trait ranges across datasets, pairwise correlations calculated on combined samples occasionally diverged from those observed within individual datasets. To address this, pairwise correlations were computed separately for each dataset, and their average was taken to provide a more robust and representative measure of trait relationships.

Furthermore, a two-step approach was adopted to determine which spectral bands most influenced trait prediction and to identify common spectral regions across traits. Inspired by Omidi et al., 2020., in the first step adjacent bands that showed pairwise Pearson correlations (Equation (4)) greater than 0.99 were merged, and 70 representative bands were obtained, with each band corresponding to a window of highly correlated wavelengths. In the second step, an ensemble machine learning framework was implemented using RF, Extreme Gradient Boosting (XGB), LinearSVR, and Ridge regression models. RF and XGB, both tree-based models, were chosen for their ability to capture complex nonlinear interactions and their robustness to noise. LinearSVR and Ridge were selected for their capacity to provide reliable linear approximations. Separate models were constructed for each trait using the 70 representative bands as predictors, and feature importance was computed based on the reduction in prediction error. Error reduction was measured using mean squared error (MSE) defined in Equation (5) in tree-based models and the absolute magnitude of regression coefficients in the linear models. The importance values were averaged across all models and five-fold cross-validation (CV), resulting in a robust assessment of the spectral regions influencing each trait.

(4) $$r=\frac{\sum \left(x_{i}-\bar{x}\right)\left(y_{i}-\bar{y}\right)}{\sqrt{\sum {\left(x_{i}-\bar{x}\right)}^{2}\sum {\left(y_{i}-\bar{y}\right)}^{2}}}$$

Where:

• r= Pearson's correlation coefficient
• $x_{i}=$ values of the x-variable
• $\bar{x}=$ mean of the x-variable values
• $y_{i}=$ values of the y-variable
• $\bar{y}=$ mean of the y-variable values

(5) $$MSE=\frac{1}{n}\sum_{i=1}^{n}{\left(y_{i}-\hat{y_{i}}\right)}^{2}$$

Where:

• $y_{i}$ ​= ​actual values
• $\hat{y_{i}}$ ​= ​predicted values
• n ​= ​number of datapoints

#### 2.3.3 Imputation model

To utilize the entire dataset for analysis, an imputation model was developed to estimate missing trait values in the Partially Labeled Set. Principal Component Analysis (PCA) was applied to the spectral data from all samples in the Partially Labeled Set and Fully Labeled Set to reduce the dimension of the hyperspectral data. The first 23 principal components (PCs), which collectively explained more than 99.99 ​% of the variance in the spectral data, were retained to serve as input features for the imputation model. This threshold was chosen to ensure that nearly all the variability in the original data was preserved while maintaining sufficient features for accurate trait prediction.

Using the Fully Labeled Set, a CNN model was trained with the 23 ​PCs as inputs and all 16 leaf traits as simultaneous outputs. CNN was selected for its ability to capture nonlinear relationships between spectral bands while leveraging local spectral patterns in the reflectance data. The model architecture consisted of a one-dimensional convolutional layer with 32 filters and a kernel size of 3, followed by batch normalization and dropout for regularization. The output from the convolutional layer was flattened and passed through two fully connected layers with 128 and 64 neurons, respectively. Both fully connected layers used the Rectified Linear Unit (ReLU) activation function and were regularized with L1 and L2 penalties to prevent overfitting. The final output layer contained 16 neurons corresponding to the leaf traits with a linear activation function.

The model was trained using log-transformed trait values to stabilize variance and improve prediction accuracy. Each trait was standardized using a separate scaler before training, and predictions were later inverse-transformed and exponentiated to recover the original scale. MSE was used as the loss function, and the Adaptive Moment Estimation (Adam) with an initial learning rate of 0.001 was employed for optimization. A five-fold CV strategy was used to evaluate model performance, ensuring that samples from all datasets were proportionately represented in the training and validation splits. For each trait, performance was assessed using R^2^, as defined in Equation (6), and the normalized root mean squared error (NRMSE), as described in Equation (7), with final metrics averaged across the cross-validation folds.

(6) $$R^{2}=1-\frac{\sum_{i=1}^{n}\left(y_{i}-\hat{y_{i}}\right)\;{\;}^{\;2}}{\sum_{i=1}^{n}{\left(y_{i}-\bar{y}\right)}^{2}}$$

(7) $$NRMSE=\frac{\sqrt{MSE}}{\max\left(y\right)-\min\left(y\right)}$$

Where:

• $y_{i}$ ​= ​actual values
• $\hat{y_{i}}$ ​= ​predicted values
• ${\bar{y}}_{i}$ ​= ​mean of the actual values
• max(y) ​= ​maximum actual value
• min(y) ​= ​minimum actual value
• n ​= ​number of datapoints

The trained model demonstrated strong predictive performance across most traits across five folds. After evaluation, the model was trained on the entire Fully Labeled Set and applied to the Partially Labeled Set to impute missing trait values.

#### 2.3.4 Trait prediction models: single- and multi-trait approaches

To prepare the spectral data for the prediction model, the approach described in Section 2.3.2 was followed, with the threshold for merging adjacent correlated bands set to 99.9 ​%, resulting in 204 bands. A higher correlation threshold was chosen for this step to retain more variance in the features, as a more significant number of samples were available for model training, and a more complex model was employed to better capture the underlying information in the data. We performed a targeted grid search over key hyperparameters, using five-fold cross-validation on the training set to select the best combination based on average validation R^2^ over all traits. The search space included:

• First LSTM layer units: 289, 128, 64
• Second LSTM layer units: 268, 128, 64
• Dropout rate: 0.16, 0.20, 0.30
• Conv1D filters (first branch): 64, 32, 128
• Conv1D filters (second branch): 32, 16, 64
• Learning rate: 5.5 ​× ​10^−4^, 1 ​× ​10^−4^, 1 ​× ​10^−3^
• Batch size: 32, 16, 64
• Number of epochs: 300, 200, 100.

For the model, two scenarios were considered: single-trait and multi-trait predictions. In both scenarios, the models share the same architecture, the only difference being in the output layer. In the single-trait scenario, the model's output layer has a single unit, with each model trained separately on individual traits. In the multi-trait scenario, the model's output layer has 16 units, corresponding to the simultaneous prediction of all traits. Each model was trained using a stratified 5-fold CV to ensure that each fold contained at least one sample from each subset of the dataset, thereby balancing representation across folds.

Given that some samples in the dataset contained imputed traits, a weighting strategy was implemented to account for the potential uncertainty introduced by the imputed values. The weights were assigned based on the average accuracy of traits in each sample. If a trait was measured, its accuracy was set to one. If a trait was imputed, its accuracy was assumed to be equal to the R^2^ value obtained for that trait in Section 2.3.3. The average accuracy across all its traits was calculated for each sample to determine a single sample-level weight. To ensure balanced contributions across the dataset, the weights were normalized by dividing each weight by the mean of all weights. This normalization ensured that the overall weight distribution remained centered around one while preserving the relative differences between fully measured and imputed samples. During model training, these weights were incorporated into the loss function to appropriately adjust the influence of samples with varying levels of data reliability.

The model architecture was designed to leverage both sequential and spatial dependencies in spectral data by integrating LSTM layers with a CNN branch. The LSTM component was chosen for its ability to process spectral bands as an ordered sequence, capturing dependencies between neighboring wavelengths. The CNN branch was included to extract local spectral features and enhance pattern recognition across the reflectance spectrum.

The LSTM branch consisted of three LSTM layers with 289, 268, and 64 units, respectively. Each LSTM layer, except the final one, was followed by a dropout layer with a dropout rate of 0.16 to mitigate overfitting. The CNN branch began with a one-dimensional convolutional layer with 64 filters and a kernel size of 3, followed by dropout regularization. A second convolutional layer with 32 filters and a kernel size of 3 was applied, followed by global average pooling and a dense layer with 64 units to extract higher-level spectral features. The outputs of the LSTM and CNN branches were combined using an additive fusion layer, integrating both sequential and local spectral patterns into a unified representation. The combined representation was passed through a fully connected layer with 50 units and ReLU activation, followed by batch normalization and dropout to stabilize the learning process. The final output layer, with a linear activation function, produced trait predictions.

The model was compiled using the Adam optimizer with a learning rate of 0.00055, and MSE was used as the loss function. Model training was optimized using callbacks, including Early Stopping, Model Checkpoint, and ReduceLROnPlateau, to improve convergence and prevent overfitting. Model performance was evaluated using R^2^ and NRMSE (as described in Equations (6), (7) and (7)) and RPD (Residual Predictive Deviation) as described in Equation (8).

(8) $$RPD=\frac{SD\;\left(y\right)}{RMSE}$$

Where:

• SD(y) ​= ​sample standard deviation of the ground-truth values in the evaluation set.

Furthermore, an additional step was performed to evaluate the model's uncertainty. This assessment was based on two metrics: (1) Dissimilarity, defined as the average Euclidean distance between each validation sample's spectral reflectance data and its five nearest neighbors in the training set, and (2) Residual, calculated as the absolute difference between the actual trait values of each validation sample and its corresponding predicted values [[51], [52], [53]].

#### 2.3.5 Sensitivity analysis of imputation quality on the multi-trait model

To assess the impact of imputation quality on multi-trait model performance, three training sets were defined. The first comprised only the samples in the Fully Labeled Set. The second included both measured samples and all imputed samples (as in Section 2.3.4). The third combined the measured samples with only those imputed samples whose mean Euclidean distance to their five nearest spectral neighbors (the dissimilarity metric from Section 2.3.4) fell below the 90th percentile of the imputed group. Each of these datasets was used to train and evaluate the multi-trait model with identical hyperparameters and the same five-fold stratified cross-validation protocol.

#### 2.3.6 Transformer-based baseline

To compare the CNN–LSTM architecture with an attention-based alternative, we implemented a compact Transformer-based baseline trained under the same protocol as Section 2.3.4. The input representation and multi-output targets are identical to those used for the CNN–LSTM models.

The network reshapes the input vector to (sequence length, 1) and applies a one-dimensional convolution (32 filters, kernel size 3, ReLU) to obtain a 32-dimensional token embedding; a trainable positional embedding of shape (sequence length, 32) is added to encode spectral order. The encoder comprises four Transformer blocks. In each block, layer normalization precedes multi-head self-attention (8 heads, key dimension 32, dropout 0.2) with a residual connection, followed by layer normalization and a position-wise feed-forward network (Dense 64 with GELU, then Dense 32) with a residual connection. The sequence representation is aggregated by global average pooling and passed through dropout (0.2), a 50-unit ReLU layer, batch normalization, and dropout (0.2) to a linear output layer with 16 units.

Training used Adam (initial learning rate 5.5 ​× ​10^−4^) with mean squared error loss, EarlyStopping (validation loss, patience 3, restore best weights), and ReduceLROnPlateau (factor 0.1, patience 3, minimum learning rate 10^−6^), for up to 300 epochs with batch size 32. Evaluation followed five-fold stratified cross-validation using the dataset label for stratification. To prevent data leakage, input standardization was fit on the training split within each fold and applied to the corresponding train and test splits; targets were scaled per trait using fold-specific scalers, and predictions were inverse-transformed with the same scalers prior to computing metrics. Per-trait R^2^ and NRMSE for this baseline are reported in Appendix F.

## 3 Results

### 3.1 Adjustment of PROSPECT-PRO Chl estimates

The performance of the PROSPECT-PRO model in estimating Chl concentrations was evaluated by comparing the model's predictions to actual measurements for 327 samples in the Fully Labeled Set. The results, visualized in Fig. 2-a and 2-b, reveal an apparent overestimation of Chl concentrations by the PROSPECT-PRO model. Fig. 2-a shows a noticeable shift between the distributions of actual and PROSPECT-PRO estimated Chl values, with the predicted values skewed higher. Fig. 2-b further illustrates this overestimation, as the best-fit regression line deviates from the 1:1 line, with a slope of 1.33 and an NRMSE of 0.41.

To correct this bias, all Chl estimates from PROSPECT-PRO were multiplied by a scaling factor of 0.75, which corresponded to the regression slope as discussed in Section 2.3.1. After this step, the adjusted PROSPECT-PRO estimates aligned closely with the actual Chl measurements. This improvement is evident in Fig. 2-c, where the predicted and actual distributions overlap more substantially. Similarly, Fig. 2-d demonstrates a near-perfect alignment with the 1:1 line, with an NRMSE of 0.17.

To assess residual variability, summary statistics were computed for the measured Chl values in Fully Labeled Set and adjusted Prospect-Pro Chl values in the Partially Labeled Set. Fully labeled samples had a mean Chl of 35.9 ​μg/cm^2^ (SD ​= ​8.6) with a 95 ​% Confidence Interval (CI) of 34.9–36.8 ​μg/cm^2^. For the partially labeled set, the mean was 27.2 ​μg/cm^2^ (SD ​= ​7.5) with a 95 ​% CI of 26.7–27.8 ​μg/cm^2^. These results indicate that the rescaling preserved an appropriate level of random uncertainty for subsequent modelling.

### 3.2 Inter-trait relationships and significance of wavelengths

**Fig. 3:**
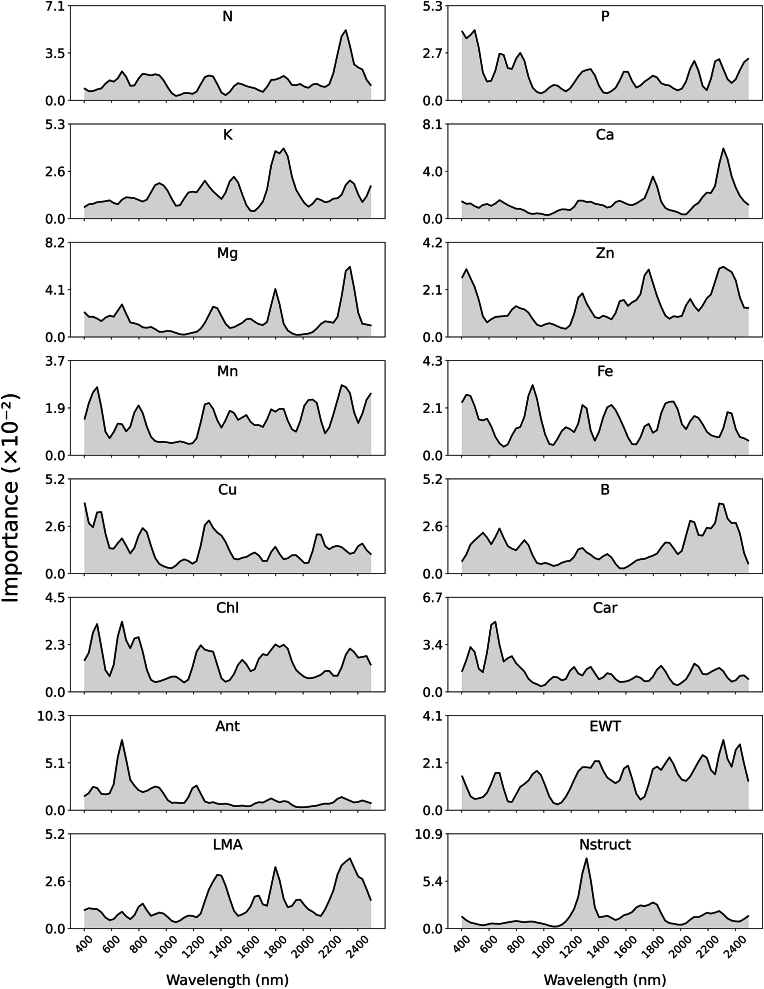
Importance of spectral bands in predicting each leaf trait.

Fig. 3 illustrates the predictive power of different spectral regions for individual leaf traits after applying a Gaussian filter for visualization purposes. N exhibits high importance around 550 ​nm and 2200 ​nm. Similarly, Chl shows pronounced importance in the visible spectrum around 450 ​nm and 550 ​nm. In contrast, EWT and LMA demonstrate increased importance in the shortwave-infrared domain, particularly around 1200 ​nm, 1700 ​nm, and 2200 ​nm.

**Fig. 4:**
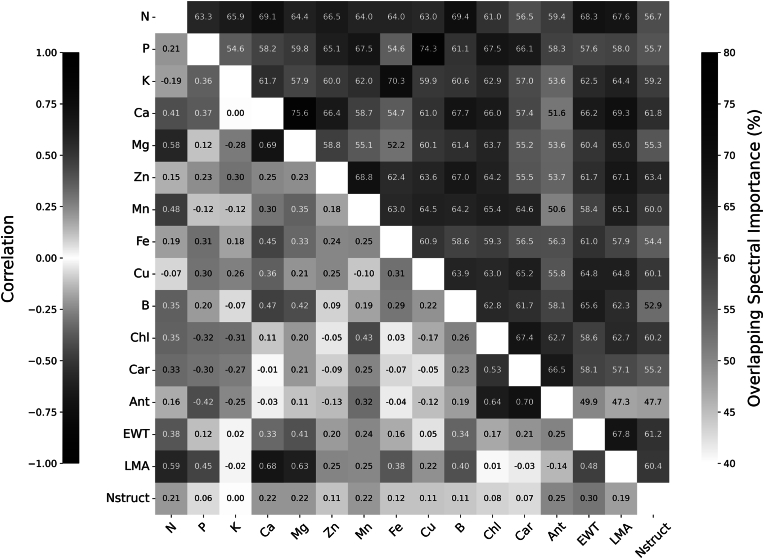
Pairwise correlations (lower left) and the percentage of shared important spectral bands (upper right) for each pair of traits.

Fig. 4 complements these findings by revealing relationships between traits through a correlation and spectral band overlap matrix. Strong positive correlations are observed between Mg and Ca (ρ ​= ​0.69) and LMA and Ca (ρ ​= ​0.68). K displays negative correlations with traits such as N (ρ ​= ​−0.19) and Chl (−0.31). Significant overlap in important spectral bands was observed between Mg and Ca (75.6 ​%). N also exhibited substantial spectral band overlaps with other traits, including P (63.3 ​%) and B (69.4 ​%). Chl also follows the same pattern, with a high percentage of overlapping important bands with traits such as P (67.5 ​%) and Ca (66.0 ​%). These results show that the traits are related both in terms of abundance and the spectral regions most informative for their prediction.

### 3.3 PCA of spectral data across all datasets

**Fig. 5:**
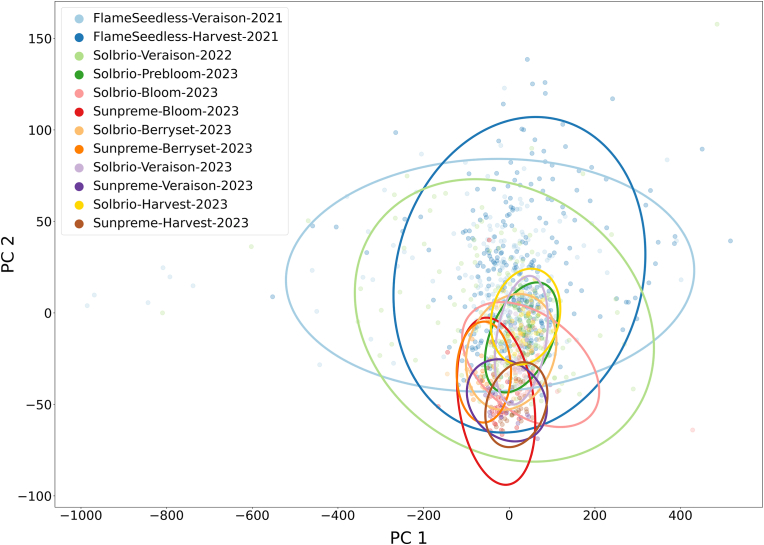
PCA of spectral data across all datasets with 95 ​% confidence ellipses.

Fig. 5 illustrates the PCA results across all datasets, specifically the distribution of samples along PC1 and PC2. Based on the PCA result, the first two PCs (PC1 and PC2) collectively explained 94.3 ​% of the total variance in the data, with PC1 accounting for 87.6 ​% and PC2 contributing 6.7 ​%. The ellipses represent 95 ​% confidence intervals for each dataset.

### 3.4 Imputation model evaluation

**Fig. 6:**
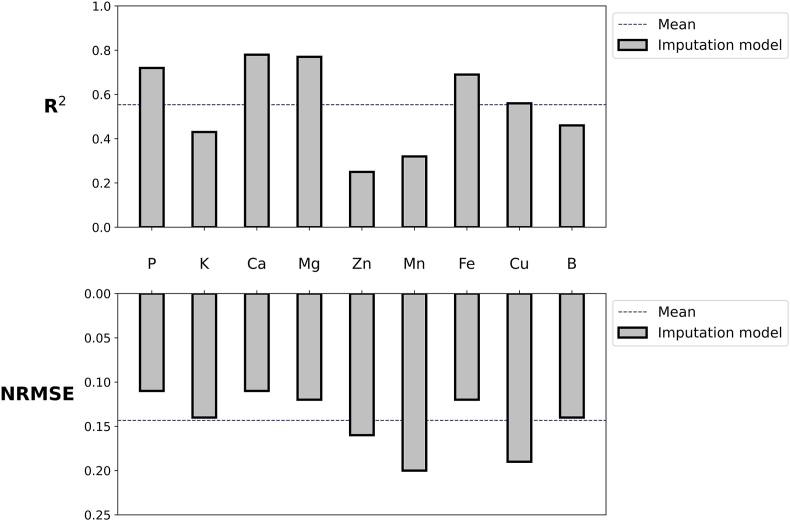
Imputation model performance for leaf traits.

The imputation model demonstrated a relatively strong predictive performance across most traits. Fig. 6 illustrates that by using the first 23 ​PCs derived from the spectral data, the trained neural network achieved varying levels of accuracy as evaluated by R^2^ and NRMSE for the nine traits requiring imputation in the Partially Labeled Set.

Nutrients such as P (R^2^ ​= ​0.72, NRMSE ​= ​0.11), Ca (R^2^ ​= ​0.78, NRMSE ​= ​0.11), and Mg (R^2^ ​= ​0.77, NRMSE ​= ​0.12) exhibited high predictive accuracy, reflecting the model's ability to capture spectral patterns associated with these traits. In contrast, some nutrients, such as Zn (R^2^ ​= ​0.25, NRMSE ​= ​0.16) and Mn (R^2^ ​= ​0.32, NRMSE ​= ​0.20), exhibited reduced performance, likely reflecting weaker spectral associations. Despite these variations, the model demonstrated overall utility in generating a complete dataset for subsequent analyses.

### 3.5 Evaluation of trait prediction models

**Fig. 7:**
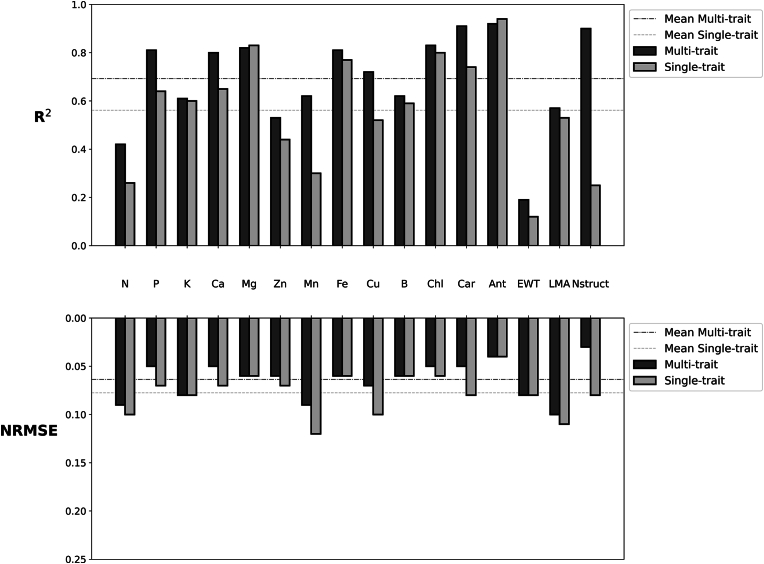
Comparing multi-trait and single-trait model performance across all traits.

The performance of the trait prediction model was evaluated using R^2^, NRMSE, and RPD under both single-trait and multi-trait modeling approaches across 16 traits. Fig. 7 presents a comparative analysis of these models, revealing that the multi-trait model consistently outperformed the single-trait model across most traits regarding predictive accuracy and precision. The values of the metrics are reported in Appendix B for both multi- and single-trait models.

The multi-trait model demonstrated substantial improvements in accuracy for predicting Mn and Nstruct, where the R^2^ increased from 0.30 to 0.62 and from 0.25 to 0.90, respectively. Similarly, Zn showed a noticeable enhancement, with an R^2^ of 0.53 in the multi-trait model, compared to 0.44 in the single-trait model. The multi-trait approach also resulted in consistently lower NRMSE values, reflecting greater prediction stability. For instance, the NRMSE of Mn reduced from 0.12 in the single-trait model to 0.09 in the multi-trait model, while Nstruct's NRMSE decreased from 0.08 to 0.03, indicating improved model reliability. Across all traits, the multi-trait model maintained a higher mean R^2^ and lower mean NRMSE, as represented by the reference lines in Fig. 7.

An uncertainty estimation framework was implemented to further evaluate model reliability, as described in Section 2.3.4. The results (Appendix C-1 and C-2) revealed a clear trend: samples with higher spectral dissimilarity tended to exhibit larger residuals, indicating areas of increased predictive uncertainty.

Notably, while our methodology primarily focused on area-based trait quantification, we also assessed the performance of the multi-trait model using mass-based results and compared them to area-based traits, as detailed in Appendix D-1 and D-2, respectively. This comparison was conducted to account for the fact that farmers are more familiar with mass-based trait assessments. Overall, area-based traits demonstrated superior predictive performance; however, notable improvements were observed in the prediction of N (R^2^ increasing from 0.42 to 0.64) and EWT (R^2^ rising from 0.19 to 0.60) when using mass-based values.

### 3.6 Comparison of predictive accuracy under different data-inclusion strategies

Applying a 90th-percentile cutoff on sample dissimilarity removed 67 low-confidence imputations, leaving 925 observations in the final training set (327 fully measured samples ​+ ​598 high-confidence imputations). Table 2 summarizes the model's R^2^ and NRMSE when trained on samples in Fully Labeled Set, Fully Labeled Set and all imputed samples, Fully Labeled set and high-confidence imputed samples.

**Table 2:** Multi-trait model performance under different data-inclusion scenarios.

Leaf Traits	Measured		Measured ​+ ​Imputed		Measured ​+ ​High Confidence Imputed	
	R^2^	NRMSE	R^2^	NRMSE	R^2^	NRMSE
N (mg/cm^2^)	0.60	0.12	0.42	0.09	0.42	0.09
P (μg/cm^2^)	0.68	0.07	0.81	0.05	0.80	0.04
K (μg/cm^2^)	0.44	0.12	0.61	0.08	0.63	0.07
Ca (μg/cm^2^)	0.75	0.10	0.80	0.05	0.83	0.06
Mg (μg/cm^2^)	0.74	0.11	0.82	0.06	0.85	0.06
Zn (μg/cm^2^)	0.16	0.10	0.53	0.06	0.38	0.06
Mn (μg/cm^2^)	0.33	0.17	0.62	0.09	0.54	0.10
Fe (μg/cm^2^)	0.63	0.10	0.81	0.06	0.80	0.06
Cu (μg/cm^2^)	0.67	0.13	0.72	0.07	0.76	0.07
B (μg/cm^2^)	0.45	0.10	0.62	0.06	0.61	0.06
Chl (μg/cm^2^)	0.54	0.11	0.83	0.05	0.83	0.05
Car (μg/cm^2^)	0.86	0.07	0.92	0.05	0.95	0.04
Ant (μg/cm^2^)	0.85	0.06	0.92	0.04	0.92	0.04
EWT (mg/cm^2^)	0.33	0.09	0.19	0.08	0.20	0.07
LMA (g/m^2^)	0.79	0.10	0.57	0.10	0.58	0.10
Nstruct	0.84	0.06	0.90	0.03	0.92	0.03

## 4 Discussion

This study evaluated the accuracy and efficiency of multi-trait versus single-trait modeling, as well as the implementation and assessment of an imputation model to enhance dataset completeness. Preprocessing steps, such as merging highly correlated spectral bands, were crucial for addressing multicollinearity and preserving valuable information. These steps allowed the model to retain essential spectral features while mitigating redundancy, ultimately improving prediction accuracy.

### 4.1 Inter-trait relationships and significance of wavelengths

Leaf spectral reflectance represents a complex signal influenced by various biochemical, nutritional, and structural traits. Understanding the relationships among these traits and their combined effects on specific spectral bands is crucial for interpreting hyperspectral data. As highlighted in Section 3.2, while some traits exhibit significant correlations, consistent with findings in other studies, others show variations.

For instance, Choudhury et al. [54] reported a high correlation between Mg and Ca (0.67) and a very low correlation between K and Ca (0.13), which aligns with our findings in Fig. 4, where Mg and Ca exhibit a correlation of 0.69, and K and Ca show no correlation. The observed strong positive correlation between leaf Mg and Ca (ρ ​= ​0.69, Fig. 4) likely stems from their shared chemical properties as divalent cations, their competitive uptake mechanisms in the soil, and their interconnected physiological roles in processes such as photosynthesis, cell wall structure, and enzyme activation [[55], [56], [57]]. This physiological relationship is reflected in their high overlapping spectral importance (75.6 ​%, Fig. 4). While the feature importance profiles (Fig. 3) reveal a distribution of important bands for both Mg and Ca across the spectrum, a closer look shows consistent importance around 1700 ​nm and 2100 ​nm regions for both traits. In contrast, the negligible correlation between K and Ca can be attributed to their distinct physiological roles and mobility within the plant. While K is highly mobile and essential for osmoregulation and enzyme activation, Ca is relatively immobile and plays a structural role in cell walls and membranes. Additionally, their potential antagonistic interactions during uptake may further explain the lack of correlation [57,58]. Despite this minimal correlation, K and Ca share a 59.1 ​% overlap in important spectral bands (Fig. 4), with notable common importance in the region around 1700 ​nm for both traits (Fig. 3). This suggests that similar spectral responses are detected in these wavelength regions despite their disparate physiological functions and mobility.

Trait relationships, while generally consistent, can exhibit significant variability depending on phenological stage, environmental conditions, sampling strategies, and population characteristics [59,60]. In Section 3.2, the results revealed a moderate negative correlation between P and the leaf pigments, including Chl, Car, and Ant, with correlation values of −0.32, −0.30, and −0.42, respectively. Similarly, K showed a similar pattern, with negative correlations of −0.31, −0.27, and −0.25 for Chl, Car, and Ant, respectively. The negative correlation of P and K with Ant is consistent with literature suggesting that nutrient deficiencies can trigger Ant accumulation as a stress response. However, the observed correlations with Chl and Car deviate from some existing studies [61,62]. This discrepancy may be due to errors in the PROSPECT-PRO estimated values for these traits. Despite these low to moderate negative correlations, the spectral band overlap between P, K, and the pigments is moderately high in Fig. 4, with P exhibiting 67.5 ​%, 66.1 ​%, and 58.3 ​% overlap with Chl, Car, and Ant, respectively. Similarly, in the important spectral bands, K shows 60.6 ​%, 62.9 ​%, and 57.0 ​% overlap with Chl, Car, and Ant. This indicates that while the physiological interactions between these nutrients and pigments can be complex and vary under different conditions, their spectral signatures still overlap significantly.

Additionally, the correlation between LMA and N, P, Ca, and Mg is positive, with values of 0.59, 0.45, 0.68, and 0.63, respectively, as shown in Fig. 4. These findings are consistent with those reported by Cherif et al. [51], where the correlations were 0.72, 0.45, 0.41, and 0.57, respectively. The positive correlations of LMA with N and P highlight their involvement in photosynthetic proteins, nucleic acids, and energy transfer, thus meeting the higher metabolic and structural demands of leaves with greater LMA [63,64]. The strong association between LMA and both Mg and Ca is further supported by the tendency of LMA to increase with leaf age [65]. Specifically, Ca, as a primarily xylem-mobile nutrient, accumulates in leaves over time due to its limited phloem mobility [66,67]. Consequently, the important spectral bands for LMA in Fig. 3 exhibit strong peaks in regions similar to Ca around 1700 ​nm and 2200 ​nm. Similarly, Mg tends to accumulate in older leaves [55,68], which also exhibits substantial overlaps in similar spectral regions with LMA.

Notably, Nstruct generally shows a moderate to low correlation with other leaf traits, indicating its relative independence from direct biochemical constituents. While leaf structure impacts the reflectance in the electromagnetic spectrum, from visible to shortwave infrared regions, it exhibits a distinct peak around 1200 ​nm, as shown in Fig. 3. Nstruct primarily affects reflectance within a specific spectral range (approximately 1000–1300 ​nm), as modeled by PROSPECT PRO [45], whereas other traits do not exhibit a similarly pronounced peak in this region.

Fig. 4 further shows a positive correlation between Chl and Car, which aligns with existing literature [69,70], while the observed positive correlation between Ant and Chl deviates from typical findings [71,72]. Despite their correlation, Chl, Car, and Ant exhibit significant peaks in their spectral importance, with Chl and Car peaking around 450 ​nm and 550 ​nm, while Ant shows a distinct peak near 570 ​nm. Although these pigments have distinct spectral effects, their spectral influence significantly overlaps, with Chl and Car sharing 67.4 ​% of their important spectral bands and Chl and Ant overlapping by 62.7 ​%. This high degree of overlap suggests that individual trait effects on spectral data cannot be fully isolated and must be considered in combination.

This complex interplay between biochemical and structural traits further underscores the challenges in separating their spectral contributions using data-driven models. While machine learning approaches can effectively leverage these relationships for trait prediction, they inherently reflect the collective influence of multiple traits rather than attributing spectral variations to a single factor. The correlations and overlapping spectral bands provide valuable insights into the potential of hyperspectral data for predicting leaf traits and also highlight the need for integrative modeling approaches that account for these interdependencies. Ensuring the robustness of hyperspectral trait prediction requires developing models that consider these interactions across varying environmental conditions and sampling contexts, ultimately improving accuracy in both ecological and agricultural applications.

### 4.2 Trait prediction models

The comparison between multi-trait and single-trait model predictions demonstrates that while single-trait models exhibit strong predictive accuracy for some individual traits, the multi-trait approach generally performs better due to its ability to leverage inter-trait relationships. Specifically, single-trait models produced slightly higher predictive accuracy for traits such as Ant and Mg, with R^2^ values of 0.94 and 0.83, respectively, compared to 0.91 and 0.82 in the multi-trait model. However, the similar NRMSE values in both approaches suggest that the practical difference is negligible. For most other traits, including N, P, Ca, Mn, Cu, Car, and Nstruct, the multi-trait model notably outperformed the single-trait models, as evidenced by higher R^2^ values.

In addition, the CNN–LSTM multi-trait architecture outperformed the Transformer-based baseline (Appendix F), likely because convolution captures local spectral structure and LSTM layers encode sequential dependencies, whereas the attention-based model is more data-hungry and less inductively biased for smooth one-dimensional spectra in our sample regime.

Compared to previous studies, the multi-trait model proposed in this study demonstrated competitive performance. For instance, Roelofsen et al. [73] reported an R^2^ of 0.46 for N and 0 for P using a PLSR model, while our model achieved comparable performance for N (R^2^ ​= ​0.42) and a significantly higher R^2^ of 0.81 for P. Similarly, Kothari et al. [74] reported R^2^ values of 0.55, 0.54, and 0.39 for mass-based K, Ca, and Mg, respectively, all of which are lower than the corresponding values of our model (0.61, 0.80, and 0.82). Likewise, their R^2^ of 0.64, 0.29, 0.29, and 0.25 for mass-based Car, Cu, Fe, and Mn are all lower than our model's R^2^ values of 0.91, 0.72, 0.81, and 0.62, further highlighting the advantages of the multi-trait approach in predicting most traits with greater accuracy. In contrast, the lower R^2^ of 0.57 for predicting LMA by the multi-trait model compared to R^2^ of 0.89 in their study may be due to the limited representation or variability of LMA values in the dataset used in this study.

For elements like Fe, Mn, Zn, and B, our model achieved R^2^ values of 0.81, 0.62, 0.53, and 0.62, respectively. Compared to Chen et al. [75], who used a PLSR model to predict mass-based Fe, Mn, Zn, and B with R^2^ values of 0.74, 0.62, 0.67, and 0.32, our approach demonstrates competitive or improved predictive performance, particularly for Fe and B.

Furthermore, the multi-trait model outperformed PROSPECT-PRO predictions, which yielded R^2^ values of −6.48, −2.08, and −6.06 for N, LMA, and EWT, respectively, indicating that simply using the mean of observed trait values would be a more accurate predictor than the estimates provided by PROSPECT-PRO. This substantial discrepancy underscores PROSPECT-PRO's limitations, particularly when applied to grapevine data. As a general-purpose model not specifically calibrated for grapevine traits, its assumptions and parameterization may not align well with the specific physiological and biochemical characteristics of grape leaves, thereby affecting its predictive capability.

The superior performance of the multi-trait model for most traits can be attributed to its ability to effectively capture trait interrelationships, as discussed in Section 4.1. This approach is particularly advantageous in agricultural and ecological contexts, where plant traits rarely function independently. By leveraging the substantial spectral overlap among traits and simultaneously accounting for their synergistic responses to environmental conditions, the multi-trait model provides a hybrid framework that enhances the accuracy and robustness of spectral trait predictions. In contrast, single-trait models primarily isolate trait-specific spectral features and do not account for trait interdependencies, potentially leading to misattribution of spectral variations. This limitation is particularly pronounced under field conditions, where dynamic environmental factors and complex trait interactions create nonlinear relationships that single-trait models often cannot effectively capture.

### 4.3 Uncertainty estimation

An essential component of this study was the incorporation of uncertainty estimation, which provided critical insights into the reliability of the model predictions. This was achieved by analyzing the dissimilarity between the spectral data of the validation and training sets and its impact on prediction errors. For both single- and multi-trait models, residuals generally increased as spectral dissimilarity increased for most traits. This trend is expected, as samples with spectral characteristics significantly deviating from the training data are more challenging for the model to predict accurately, resulting in higher residuals. This relationship is demonstrated by the positive Ordinary Least Squares (OLS) regression slopes observed for most traits in Appendix C-1 and C-2.

However, this pattern was not consistent across all traits. For instance, traits such as Mn and K exhibited negative OLS slopes in the multi-trait uncertainty plot (Appendix C-1), while Zn and B had slopes close to zero, indicating relatively high residuals even for samples with high spectral similarity. This variability in model performance highlights potential areas for improvement, such as acquiring additional training data to represent these traits better and reduce prediction errors. Furthermore, when comparing multi-trait and single-trait models, single-trait models showed higher residuals at the same level of spectral dissimilarity across most traits. This indicates greater uncertainty in single-trait predictions, underscoring the advantages of the multi-trait approach.

The implications of these findings extend beyond grapevine analysis and suggest valuable applications for the broader field of precision agriculture. The multi-trait approach provides a scalable framework for trait prediction that could be adapted for various crops and complex trait datasets. The ability to accurately and efficiently predict interrelated plant traits could enhance crop health monitoring, optimize nutrient management, and ultimately improve crop yield and quality in various agricultural contexts. Specifically, in viticulture, where maintaining optimal nutrient balance and physiological health is critical for fruit quality, multi-trait models may offer vineyard managers more precise and holistic insights into vine health, enabling timely and targeted interventions.

### 4.4 Influence of high-confidence sample selection on model accuracy

Removing the 90th-percentile lowest-confidence imputations had very little effect on overall model performance: the “measured ​+ ​imputed” and “measured ​+ ​high confidence imputed” scenarios produced nearly identical R^2^ and NRMSE values for most traits. Zn and Mn were the only exceptions, showing the largest drop in accuracy when the low-confidence imputed samples were removed. This likely reflects the inherently weaker or more subtle spectral signatures of Zn and Mn, because samples near the confidence cutoff still carry valuable variance for these traits, so excluding them reduces the model's ability to learn those delicate patterns.

In contrast, comparing the “measured” versus “measured ​+ ​imputed” scenarios revealed more pronounced differences. For most traits, adding imputed samples boosted performance by exposing the network to a broader range of spectral-trait relationships. Unexpectedly, however, N, EWT and LMA, all of which were fully measured and never imputed, showed better accuracy when only the measured data were used. We hypothesize that although these traits had complete measured coverage, introducing additional imputed cases for the other traits subtly shifted the joint spectral distribution during training. Because the network learns all outputs jointly, even unmixed traits can suffer if the overall data distribution drifts. In essence, the imputed records, drawn from other datasets, may have introduced slight biases or noise that counteracted the benefits of a larger sample size for N, EWT and LMA.

Overall, these results underscore that while imputations can enhance model robustness for many traits, their inclusion must be managed carefully. Filtering out low-confidence imputations has minimal downside for most traits, but trait-specific handling of synthetic samples may be necessary to avoid unintended distributional shifts, especially for traits with strong measured signals or inherently weak spectral signatures.

## 5 Conclusions

This study highlights the potential of hyperspectral sensing and advanced modeling techniques to enhance nutrient monitoring and management in precision viticulture. By leveraging spectral data from 400 to 2500 ​nm and employing single-trait and multi-trait models, the research demonstrates the advantages of multi-trait approaches in capturing interdependencies among traits. Using a hybrid CNN-LSTM network, the multi-trait model outperformed single-trait models in predictive accuracy and computational efficiency for various traits. The study also showed the effective integration of spectral data with imputation models to address missing values, thereby enabling the use of a complete dataset for model training and validation. These findings underscore the importance of multi-trait modeling for capturing complex physiological and biochemical relationships in grapevine leaves, paving the way for more reliable and scalable nutrient monitoring solutions.

Despite its successes, this study has several limitations that underscore opportunities for further refinement. One significant limitation is the reliance on the PROSPECT-PRO radiative transfer model for estimating traits such as Chl, Ant, and Car. Although PROSPECT-PRO has been widely utilized in studies lacking ground truth data and is effective for general assessments, its application across multiple crop types can introduce biases, mainly when applied to grapevine data. Additionally, the bias in PROSPECT-PRO's Chl prediction might not be linear as assumed in this study, suggesting that simple scaling may not adequately correct the estimates. This possibility of non-linear biases necessitates further research. For other traits such as Car, Ant, and Nstruct, where we also used PROSPECT-PRO predictions, we assumed that biases were linear and could be corrected by applying a scale factor to the estimated values. This assumption presumes a straightforward relationship that simplifies adjustments but requires further validation. Additional data collection for these traits is essential to understand and correct these biases thoroughly.

Another limitation involves the imputation model used to handle missing data for nutrients such as P, K, Ca, Mg, Zn, Mn, Fe, Cu, and B. While we attempted to mitigate uncertainty by assigning lower weights to samples with higher imputation uncertainty, this approach may not fully resolve the challenges associated with imputing missing data. Exploring alternative imputation models or methods could provide more robust solutions and further improve the reliability of the dataset, especially for traits with higher variability or weaker spectral signals.

The continued refinement of spectral sensing and modeling frameworks holds significant promise for advancing precision viticulture. Addressing the identified limitations and pursuing innovative research directions could enhance the potential for scalable, non-destructive, and highly accurate nutrient monitoring systems, which may ultimately contribute to more sustainable vineyard management.

## Author contributions

Parastoo Farajpoor: Methodology, Data collection, Software, Formal analysis, Validation, Visualization, Investigation, Writing original draft. Alireza Pourreza: Conceptualization, Methodology, Supervision, Review and editing of manuscript, Funding acquisition, Resources. Mohammadreza Narimani: Data collection, Review and editing of manuscript. Ashraf El Kereamy: Data collection, Review and editing of manuscript. Matthew Fidelibus: Data collection, Review and editing of manuscript.

## Data availability

The model and sample data is available at https://www.digitalaglab.com/LeafMonitorApp.

## Declaration of competing interest

The authors declare the following financial interests/personal relationships which may be considered as potential competing interests. Alireza Pourreza reports financial support was provided by National Institute of Food and Agriculture. If there are other authors, they declare that they have no known competing financial interests or personal relationships that could have appeared to influence the work reported in this paper.

## Figures and Tables

**Table:** Evaluation metrics (R^2^ and NRMSE) of the imputation model across all traits

Traits	R^2^	NRMSE
N (mg/cm^2^)	0.61	0.14
P (μg/cm^2^)	0.72	0.11
K (μg/cm^2^)	0.43	0.14
Ca (μg/cm^2^)	0.78	0.11
Mg (μg/cm^2^)	0.77	0.12
Zn (μg/cm^2^)	0.25	0.16
Mn (μg/cm^2^)	0.32	0.20
Fe (μg/cm^2^)	0.69	0.12
Cu (μg/cm^2^)	0.56	0.19
B (μg/cm^2^)	0.46	0.14
Chl (μg/cm^2^)	0.54	0.14
Car (μg/cm^2^)	0.89	0.07
Ant (μg/cm^2^)	0.77	0.10
EWT (mg/cm^2^)	0.35	0.15
LMA (g/m^2^)	0.87	0.09
Nstruct	0.78	0.09

**Appendix B:** Evaluation metrics (R^2^, NRMSE and RPD) of single-trait and multi-trait models across all traits

Traits	R^2^		NRMSE		RPD	
	Single-trait	Multi-trait	Single-trait	Multi-trait	Single-trait	Multi-trait
N (mg/cm^2^)	0.26	0.42	0.10	0.09	1.28	1.32
P (μg/cm^2^)	0.64	0.81	0.07	0.05	1.47	2.22
K (μg/cm^2^)	0.60	0.61	0.08	0.08	1.39	1.55
Ca (μg/cm^2^)	0.65	0.80	0.07	0.05	2.11	2.32
Mg (μg/cm^2^)	0.83	0.82	0.06	0.06	2.28	2.54
Zn (μg/cm^2^)	0.44	0.53	0.07	0.06	1.36	1.48
Mn (μg/cm^2^)	0.30	0.62	0.12	0.09	1.36	1.62
Fe (μg/cm^2^)	0.77	0.81	0.06	0.06	2.24	2.41
Cu (μg/cm^2^)	0.52	0.72	0.10	0.07	1.39	1.88
B (μg/cm^2^)	0.59	0.62	0.06	0.06	1.31	1.55
Chl (μg/cm^2^)	0.80	0.83	0.06	0.05	1.69	2.44
Car (μg/cm^2^)	0.74	0.92	0.08	0.05	4.06	3.31
Ant (μg/cm^2^)	0.94	0.92	0.04	0.04	4.56	3.61
EWT (mg/cm^2^)	0.12	0.19	0.08	0.08	1.06	1.12
LMA (g/m^2^)	0.53	0.57	0.11	0.10	1.51	1.51
Nstruct	0.25	0.90	0.08	0.03	1.53	3.14

**Appendix C-1:**
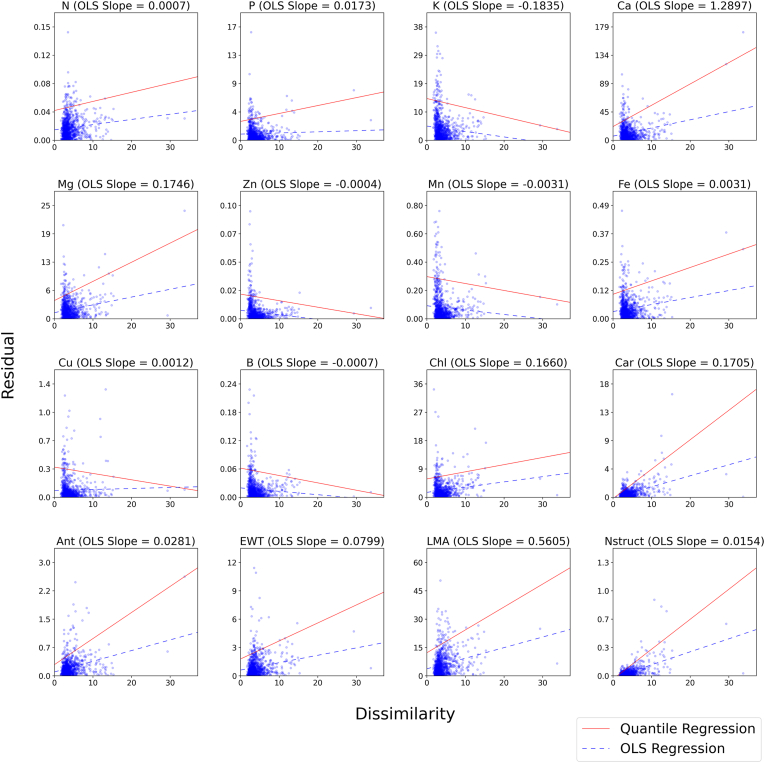
Quantitative analysis of spectral dissimilarities and trait prediction residuals in multi-trait and single-trait models

**Appendix C-2:**
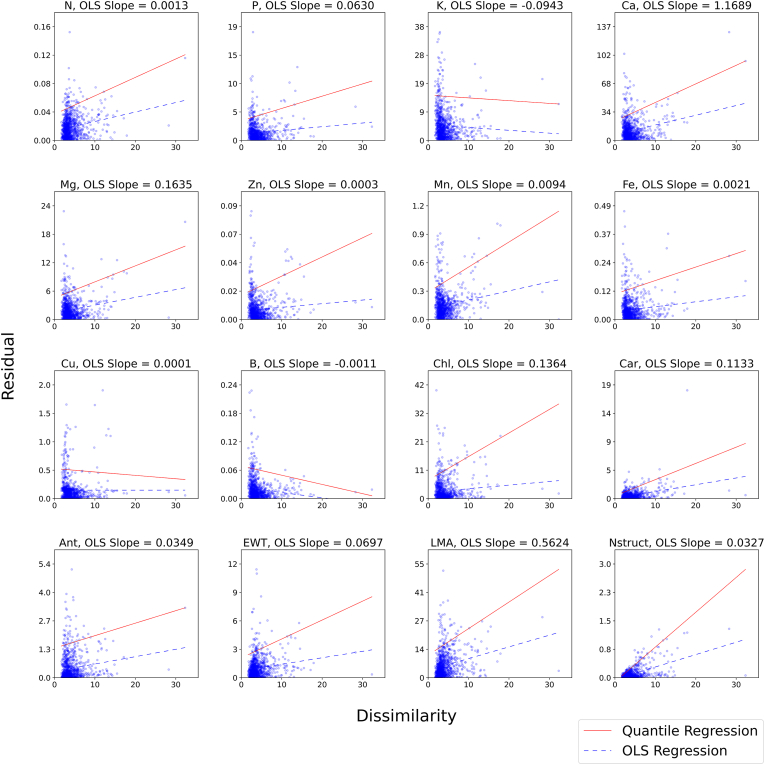
Quantitative analysis of spectral dissimilarities and trait prediction residuals in multi-trait model

**Appendix D-1:** Evaluation metrics (R^2^ and NRMSE) of multi-trait model on mass-based traits

Traits	R^2^	NRMSE
N (mg/cm^2^)	0.64	0.10
P (μg/cm^2^)	0.76	0.06
K (μg/cm^2^)	0.52	0.10
Ca (μg/cm^2^)	0.54	0.08
Mg (μg/cm^2^)	0.52	0.07
Zn (μg/cm^2^)	0.54	0.08
Mn (μg/cm^2^)	0.57	0.07
Fe (μg/cm^2^)	0.42	0.08
Cu (μg/cm^2^)	0.70	0.07
B (μg/cm^2^)	0.41	0.08
Chl (μg/cm^2^)	0.71	0.07
Car (μg/cm^2^)	0.79	0.07
Ant (μg/cm^2^)	0.83	0.06
EWT (mg/cm^2^)	0.60	0.09
LMA (g/m^2^)	0.55	0.10
Nstruct	0.82	0.04

**Appendix D-2:**
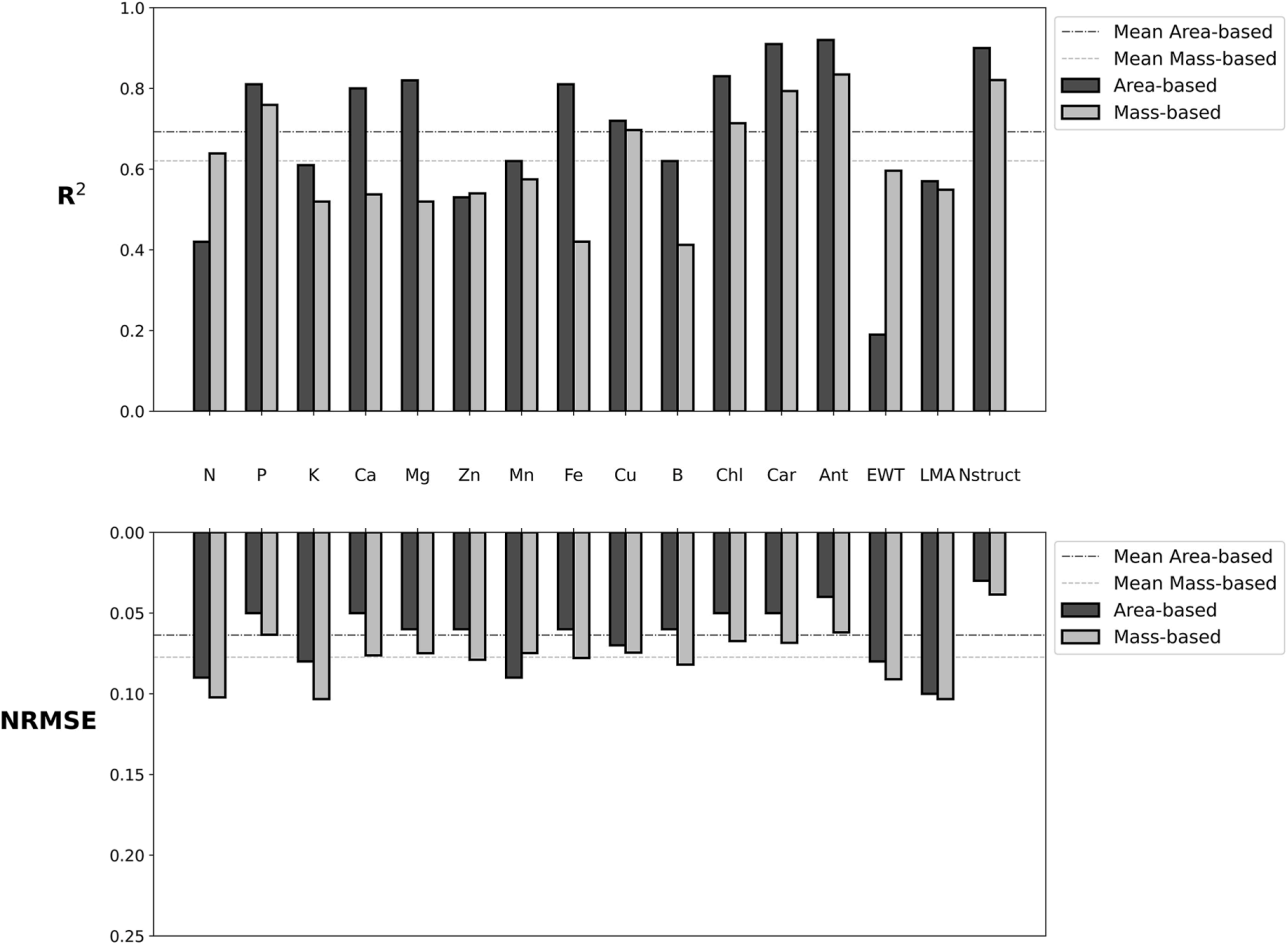
Comparing the performance of the multi-trait model on area-based versus mass-based traits

**Appendix E:**
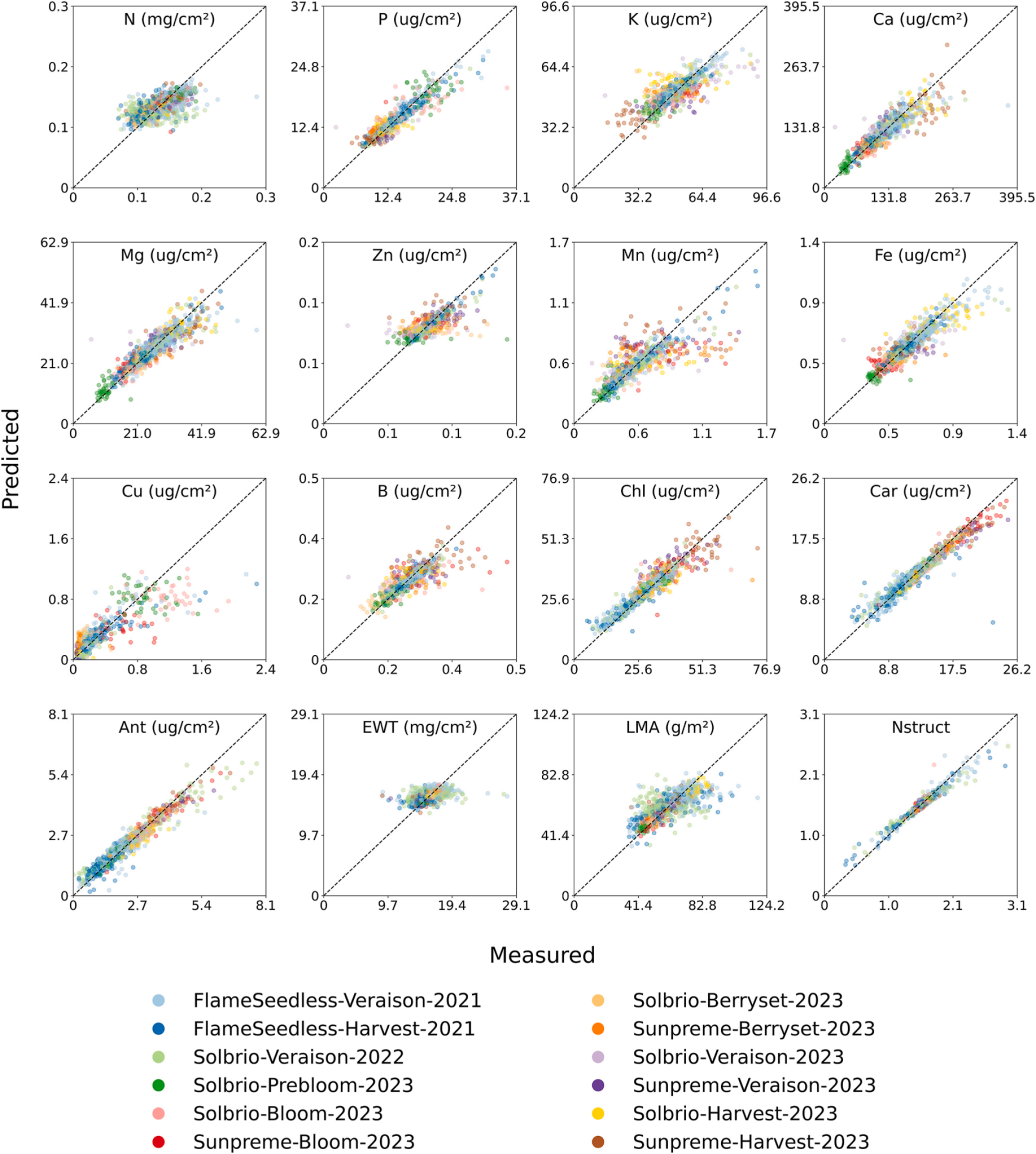
Comparative analysis of multi-trait predicted vs. measured trait values across different datasets

**Appendix F:** Evaluation metrics (R^2^ and NRMSE) of Transformer-based multi-trait model

Traits	R^2^	NRMSE
N (mg/cm^2^)	0.20	0.11
P (μg/cm^2^)	0.58	0.07
K (μg/cm^2^)	0.35	0.10
Ca (μg/cm^2^)	0.54	0.08
Mg (μg/cm^2^)	0.58	0.09
Zn (μg/cm^2^)	0.42	0.07
Mn (μg/cm^2^)	0.51	0.10
Fe (μg/cm^2^)	0.62	0.08
Cu (μg/cm^2^)	0.40	0.11
B (μg/cm^2^)	0.41	0.08
Chl (μg/cm^2^)	0.72	0.07
Car (μg/cm^2^)	0.79	0.09
Ant (μg/cm^2^)	0.63	0.09
EWT (mg/cm^2^)	0.09	0.08
LMA (g/m^2^)	0.36	0.12
Nstruct	0.77	0.04
